# Clinical Presentation of Retinoblastoma in Ethiopia: A Case of Jimma University Medical Center Pediatric Oncology Unit, Southwest Ethiopia

**DOI:** 10.4314/ejhs.v31i5.7

**Published:** 2021-09

**Authors:** Diriba Fufa Hordofa, Kumale Tolesa Daba, Aemero Abateneh Mengesha

**Affiliations:** 1 Jimma University, Département of Pediatrics, Jimma, Ethiopia; 2 Jimma University Département of ophthalmology. Jimma, Ethiopia; 3 Bahir Dar University Département of Ophthalmology, Bahir Dar, Ethiopia

**Keywords:** Retinoblastoma, leukocoria, orbital disease

## Abstract

**Background:**

Retinoblastoma (RB) is one of the most curable childhood cancers if early detected and treated. Late presentation complicates the management of RB results in dismal treatment outcome. Objective: The aim of this study is to report the clinical presentation pattern of retinoblastoma patients seen at Jimma University Medical center (JUMC).

**Methods:**

The study was a retrospective review of retinoblastoma cases managed at JUMC between August 2016 and July 2020.

**Results:**

Among pediatric oncology cases registered retinoblastoma, accounting 8.5 % (36/423) of all childhood cancer patients in the study period, 29 (90.6%) of them had unilateral retinoblastoma and 3(9.4%) of them had bilateral retinoblastoma. The average age at presentation for bilateral and unilateral retinoblastoma patients was 17 (range 3–30) months and 37.5 months (range 8–84) respectively. The first symptom observed by the family was leukocoria in 21 (65.6%) of the patients but 24(75%) of the patients presented with advanced stage (proptosis and fungating orbital mass) of the disease. The longest and the shortest lag time of presentation from the first symptom was 17 months and 2 weeks respectively with the mean lag time of 1.4 months in bilateral and 6 months in unilateral cases. Clinically, the majority of the eyes 24/35(68.6%) were extraocular tumors involving orbital tissues at presentation.

**Conclusion:**

Most of retinoblastoma patients presented at advanced stage of the disease and presented very late after the family observed the disease. Early detection strategies must be designed by the government and responsible stakeholders in mitigating the effects of late presentation.

## Introduction

Retinoblastoma (RB) is a highly malignant tumor of the eye and the most common intraocular cancer of childhood with the worldwide incidence of one case per 15,000–20,000 live births, corresponds to about 9,000 new cases every year globally (1).

More than 95% of children survive from retinoblastoma in developed nations where as the survival can be as low as 6.8% in less developed countries. Early detection, prompt treatment while the tumor is still localized to the eye and multidisciplinary approach are the reasons for the success of retinoblastoma intervention in developed countries in which the focus for management is sight preservation. On the contrary late presentation and recognition is almost the rule in less developed countries at which stage management of the cases becoming very challenging, and in most cases the focus is mainly life preservation (2).

Earlier studies in developing countries including Nigeria and Senegal reported the patient presented at advanced stage of the disease and presented very late after observing first symptoms by the family, contributed for delayed detection and treatment which causes poor treatment outcome of the patient. Other factors affecting patient outcome includes, as Paucity of staff, absence of expert centers, shortage of anticancer drugs, lack of financial resources, and underlying malnutrition (2,3,4). Understanding the clinical presentation of retinoblastoma patients in the study area is a stepping stone to design early detection and management strategy of retinoblastoma in Ethiopia. Our aim is to report the pattern of clinical presentation of retinoblastoma based on single institution in a newly opened pediatric oncology unit in Jimma medical center, south west of Ethiopia.

To our knowledge this is the first report about the presentation of retinoblastoma in South Western Ethiopia. This will serve in identifying challenges to proper treatment of retinoblastoma in Ethiopian patients and hopefully design solutions to such challenges.

## Materials and Methods

Jimma Medical Center is a government teaching and referral hospital serving more than 25million catchment population; it is located in Oromia regional state, Jimma Town, 355km south west of the capital, Addis Ababa. The Center is a general hospital having 800 beds and pediatrics department is one of the major departments having 120beds. The department has general wards and as well as specialty units like pediatric intensive care unit (ICU), Neonatal ICU and pediatric oncology. The hospital also has relatively strong ophthalmology department with Pediatric ophthalmology and ophthalmic plastic subspecialty services. Before the establishment of Pediatric oncology unit (POU) at Jimma University Medical Center (JUMC) in August 2016, enucleation and orbital exenteration were the only treatment modalities provided for retinoblastoma patients at our ophthalmology department.

The pediatric oncology unit was established in August 2016 with the collaboration of Jimma University and The ASLAN project, a USA based non-for-profit Organization. Currently the unit has 20 beds, running with the team of one full time pediatrics Hematologist/oncologist, four pharmacists, ten nurses, one data clerk and one social worker. There is no radiotherapy service in the hospital.

The study was a retrospective review of retinoblastoma patients managed at the JUMC between August 2016 and May 2020. All retinoblastoma patients treated at JUMC- POU during the study period were included in the study.

Listings were retrieved from the excel register of the JUMC- POU to identify those treated during the study period. From a total of 423 pediatric oncology cancer cases registered on the excel spread sheet of the unit during the study period of which 36 patients were retinoblastoma cases. Charts were obtained and reviewed for 32 patients only.

The data, including age and sex of the patient, first Symptom observed by the family, duration of first symptom at presentation, clinical signs at presentation, laterality of the disease, diagnostic imaging studies used to diagnose the disease, histopathology findings, stage of the disease according to International Retinoblastoma Staging System and primary treatment given for the patient were collected. The data entered and analyzed using Microsoft excel work sheet version 10.0. The result presented in frequency distribution tables.

Ethic clearance was obtained from Jimma university health institute Institutional Review Board (IRB) with the reference number of IHRPGD/436/2018. Confidentiality was kept by excluding patient identifier like name and medical record number from data collection. The study was conducted in accordance with the tenets of the declaration of Helsinki.

## Results

A total of 36 patients were diagnosed to have retinoblastoma at JUMC_POU, consisting 8.5 %( 36/423) of all childhood cancer patients seen at the unit during the study period. But the charts of 4 patients were not found and excluded from the analysis.

Our study showed a total 35 eyes were diagnosed to have retinoblastoma in 32 patients. Females were the most common gender, which accounts 22 (68.8%), with female to male ratio of 2.2:1. Our study also showed 29(90.6%) of the patients had unilateral retinoblastoma with the average age at presentation of 37.5months (range 8–84months) and 17months (range 3–30months) months for bilateral retinoblastoma. The most common symptoms first noted by the families were leukocoria in 21(65.6%) followed by red eye 6 (18.5%) and proptosis 5 (15.6%). The most common clinical signs presented to the clinic were proptosis 20(62.5%) followed by leukocoria 7 (21.9%) and fungating orbital mass 4 (12.5%). The maximum delay of patients to seek medical care after the family observed first symptom was 17 months and the shortest delay time was 2 weeks with the mean time of delay for bilateral cases was 1.4 months and 6 months for unilateral cases (Table 1).

**Table 1:** Charactrestics of children (n=32) diagnosed with retinoblast at Jimma Univesity medical center from August 2016 to July 2020, Jimma, Ethiopia

*Variable*	*Category*	*No (%)*
Gender	Male	10 (31.2)
	Female	22 (68.8)
Age in months, Mean (range)	Unilateral (n=29)	37.5 (8–84months)
	Bilateral (n=3)	17 (3–30months)
First symptoms	Leukocoria	21(65.6)
	Proptosis	5 (15.6)
	Red eye	6 (18.8)
Presenting clinical signs	Proptosis	20(62.5)
	Leukocoria	7(21.9.3)
	Fungating mass	4(12.5)
	Ocular inflammation	1(3.1)
Diagnostic Lag time in months, Mean(range)	Unilateral	6 (0.5–17)
	Bilateral	1.4 (0.5–3)
Diagnostic investigation used	Ultrasound(B-scan)	27(84.4)
	CT scan	2(6.2.3)
	No imaging found in their record	3(9.4)

In addition to clinical finding, diagnostic imaging showed intraocular mass with calcification was used to confirm the likelihood of retinoblastoma for which B-scan Ultrasound done in 27 (84.4.7%) patients and CT scan for 2 patients. For 3 patients the imaging record was not found in their charts (Table 1).

Clinically, majority of the eyes 24 (68.6%) were presented with extraocular tumor involving orbital tissues followed by advanced intraocular tumor 11 (31.4%). The stage of the disease according to International Retinoblastoma Staging System were stage I 5 (14.3%), stage II 2 (5.7%), stage III 11(31.4%) and stage IV 6(17.2%). It was not possible to stage in 11 (31.4%) eyes since the pathology report was not found in their chart (Table 2).

**Table 2:** Clinical finding and International retinoblastoma stage of children eyes (n=35) diagnosed with retinoblast at Jimma Univesity medical center from August 2016 to July 2020, Jimma, Ethiopia

*Variables*	*Right eye* *N (%)*	*Left eye* *N (%)*	*Total* *N (%)*
Clinical finding			
Advanced Intraocular tumor	4(11.4)	7(20.0)	11(31.4)
Exteraocular tumor involving orbital tissue	12(34.3)	12(34.3)	24(68.6)
International Retinoblastoma Staging system			
Eye enucleated. Completely resected (stage 1)	3(8.6)	2(5.7)	5(14.3)
Eye enucleated, microscopic residual tumor (stage 2)	2(5.7)	0(0.0)	2(5.7)
Orbital or regional lymph node involvement (stage 3)	4(11.4)	7(20.0)	11(31.4)
Metastatic disease (stage 4)	3(8.6)	3(8.6)	6(17.2)
Unknown	4(11.4)	7(20.0)	11(31.4)

## Discussion

Retinoblastoma is the most common childhood intraocular tumor which accounts for 1:15,000–20,000 live births (1).

This retrospective study shows higher burden of retinoblastoma 8.5% among all pediatric oncology cases compared to 4.8% in Sudan and 3% mentioned by World Health Organization (1, 5). However, it is consistent with the findings of Stiller and Parkin who explained the higher burden in Africa, India and Native Americans (6). Although the effect of ethnic background and socioeconomic factors is not well understood, some studies suggest some association with poverty and low levels of maternal education (7,8).

The incidence in female is higher than male which contradict with the already known epidemiological data regarding retinoblastoma (8–11). Since the study is small scale and a hospital-based study, this doesn't show the magnitude in the population. The average age at presentation stated in this study is similar to other previous studies done which is less than 2 years of age (1, 9).

The most common presenting sign found in this retrospective data was (proptosis 20 (62.5%) which is consistent with findings from other developing countries (2, 3,4). Majority of eyes were diagnosed with extra ocular tumor 24 (68.8%). According to International Retinoblastoma Staging system majority were Stage III 11(33.3%). This finding is consistent with most studies in developing countries, where there is a delay in presentation (2, 3,4, 12). A study done in Ethiopia at Menelik II hospital also showed proptosis to be the commonest presenting sign (53.7%) followed by leucocorea (22%) (13).

The most common first symptom mentioned by parents in the current study was leukocoria (65.6%). Unilateral retinoblastoma was more common (90.6%). This is comparable to the previous studies and text books (1, 9,13, 14). This study also revealed there was a mean patient delay time to seek medical care from the date of observation of the first symptom (1.4 months for bilateral and 6months for unilateral cases). This finding is similar to previous studies done in Ethiopia Menilik II Hospital, Alexanderia, and Malaysia (12–14). This is a very sad situation where parents noted the disease at the earlier stage, leukocoria, but when the first manifestation is other than leukocoria, such as strabismus, they bring children at a later stage where the mortality is very high and the treatment as well as the cure is very difficult. This is pronounced in one study which revealed a significantly increased diagnostic delay in those presenting with squint rather than leucocoria (15). As a study in Alexanderia showed the delay in presentation is significantly associated with advanced disease in both unilaterally and bilaterally affected children, which in turn makes the management more difficult (14). This study signifies that there might be lack of knowledge among the society and primary care physicians in the periphery. Further study is needed to confirm the society's and primary care physicians' level of awareness and practice. Furthermore, studies suggested that Retinoblastoma educational and public awareness campaigns increase referrals, decrease rates of advanced disease, and improve outcomes in developing countries (16).

The major limitation of this study was small number of the study subjects in which results and conclusions of the study should be interpreted with caution. In addition, some pathology results were not found in the patient charts and some charts were lost.

This retrospective study was the first of its kind in South West Ethiopia which shows incidence rate of 8.5% of all childhood cancer patients seen at JUMC Pediatric oncology center in the first four years of establishment. Retinoblastoma was unilateral in most of the cases presented in this study. The most common presenting sign was proptosis. The mean age at presentation was 17 months for bilateral and 37.5 months for unilateral cases of retinoblastoma. Late presentation was the most significant finding in this study which strongly impacts prognosis of the disease. Retinoblastoma prevention strategy must be designed by government and other stakeholders focusing on early detection of the disease in combating this life threatening tumor.
